# The Interface of Syntax with Pragmatics and Prosody in Children with Autism Spectrum Disorders

**DOI:** 10.1007/s10803-016-2811-8

**Published:** 2016-05-21

**Authors:** Arhonto Terzi, Theodoros Marinis, Kostantinos Francis

**Affiliations:** Department of Speech and Language Therapy, Technological Educational Institute of W. Greece, Meg. Alexandrou 1, Koukouli, 26334 Patras, Greece; School of Psychology and Clinical Language Sciences, University of Reading, Reading, UK; Second Psychiatric Department, National and Kapodistrian University of Athens, Athens, Greece; Kuwait Centre for Mental Health, Shuwaikh, Kuwait

**Keywords:** Clitic pronouns, Focus, Clitic left dislocation, Interfaces, Syntax, Discourse/pragmatics, Prosody

## Abstract

In order to study problems of individuals with Autism Spectrum Disorders (ASD) with morphosyntax, we investigated twenty high-functioning Greek-speaking children (mean age: 6;11) and twenty age- and language-matched typically developing children on environments that allow or forbid object clitics or their corresponding noun phrase. Children with ASD fell behind typically developing children in comprehending and producing simple clitics and producing noun phrases in focus structures. The two groups performed similarly in comprehending and producing clitics in clitic left dislocation and in producing noun phrases in non-focus structures. We argue that children with ASD have difficulties at the interface of (morpho)syntax with pragmatics and prosody, namely, distinguishing a discourse prominent element, and considering intonation relevant for a particular interpretation that excludes clitics.

## Introduction

Until recently, research on the language of individuals with Autism Spectrum Disorders (ASD) has addressed several domains of language, including phonology and the lexicon (Rapin et al. 2009; Rescorla and Safyer 2013), with pragmatics and prosody being of particular importance, as this is where the most easily observable problems have been encountered throughout the autism spectrum (McCann and Peppe 2003; Tager-Flusberg 1999). In recent years a growing body of research has begun to investigate the (morpho)syntax of individuals with ASD with some initial studies revealing that certain aspects of it are not as intact as first believed. For example, Roberts et al. (2004) investigated the production of Tense morphology (3rd person singular—*s* and past tense—*ed*) in English-speaking children with ASD between the age of 5 and 15. The study showed that the children with ASD who scored low on general language tasks, hence, were classified as language impaired, had difficulties with Tense inflection, showing high rates of omission of tense morphemes. This was not the case for children with ASD who were not language impaired. In what may be considered more of a study in syntax proper, Perovic et al. (2013a, b) investigated the reference of personal object pronouns and reflexive pronouns of English-speaking children with ASD between the ages of 6 and 18. These studies showed that language impaired children with ASD had difficulties in the interpretation of reflexive pronouns. The majority of studies addressing the (morpho)syntax of children with ASD, including the above, investigated English-speaking children; therefore, it remains unclear whether the difficulties attested hold across languages. To address this issue it is necessary to investigate (morpho)syntactic abilities in ASD across languages.

With this in mind, Terzi et al. (2014) investigated the acquisition of reflexive pronouns, object clitic pronouns, and object strong pronouns, along with passive sentences, in 20 Greek-speaking children with ASD (mean age: 6;8) and their language-matched typically developing (TD) controls of similar chronological age. The children with ASD had non-verbal abilities within the norms, therefore, they were characterized as high-functioning. Their verbal abilities were also within the norms. The results showed that the children with ASD did not have any difficulties with reflexive and strong pronouns and performed similarly to TD children on passives. However, they had subtle difficulties with clitic pronouns in both comprehension and production. In particular, when the children with ASD erred on the reference of object clitic pronouns they reversed the thematic roles of the two participants/noun phrases of the sentence. When they erred on the production of object clitics, they produced the corresponding noun or omitted the object entirely. The authors did not offer an explanation for this behavior as their major concern was to establish the profile of Greek-speaking children with ASD on the areas of grammar addressed in the studies by Perovic et al. (2013a, b) for English, being particularly intrigued by the difficulties of the English-speaking children with autism on the reference of reflexive pronouns. Other studies targeting the (morpho)syntax of individuals with ASD in languages beyond English are the ones by Su et al. (2014) and Zhou et al. (2015). Su et al. investigated the comprehension of the *wh*-words ‘what’ and ‘who’ within appropriate sentences, administered to 28 Mandarin-speaking children with ASD who also had verbal and non-verbal abilities within the norms. These *wh*-words in Mandarin may convey a question or a statement interpretation, depending on the intonation on the *wh*-word. For example, when the Mandarin sentence ‘Monkey not buy wh-word fruit’ is used with level intonation on the *wh*-word, it is interpreted as ‘The monkey did not buy any fruit’; in contrast, when used with rising intonation on the *wh*-word, it is interpreted as ‘What fruit did the monkey buy?’. The results revealed that older children with ASD (mean age: 11;7) and the typically developing controls were accurate in the comprehension of both question and statement interpretations, but younger children with ASD (mean age: 6;6) had difficulties in the interpretation of sentences with *wh*-words as statements. Zhou et al. investigated the perfective aspect morpheme of verbs in 59 4–6-year-old Mandarin-speaking children with ASD with non-verbal abilities within the norms and MLU one year below that of their typically developing controls. The study showed that children with ASD produced target perfective aspect significantly less often than age matched, IQ matched, and language matched TD controls.

A common denominator of the above studies is that the difficulties with (morpho)syntax in high-functioning individuals with ASD, are neither severe nor present across a large number of phenomena. This raises the question of whether such difficulties result from deficits within (morpho)syntax or from deficits in pragmatics and/or prosody which affect structures that are at the interface of (morpho)syntax with pragmatics and/or prosody. The interpretation of reflexive pronouns does not relate to pragmatics or prosody. Therefore, it is not surprising that Perovic et al. (2013a) argued for a syntactic deficit in the reference of English reflexive pronouns. Note, however, that the population identified with this problem were language impaired children the majority of who also scored low on general non-verbal abilities. The authors did not further distinguish ASD children on the basis of their non-verbal abilities, but included a discussion on the potential impact of non-verbal abilities on verbal abilities (Perovic et al. 2013a). Interestingly, in a more recent study, Janke and Perovic (2015) do not detect problems with reflexives (nor with control structures) in a new pool of English-speaking children, all of which were high-functioning. Zhou et al. (2015), on the other hand, argue that the deficit in perfective aspect of Mandarin-speaking children with ASD is not a (morpho)syntactic deficit per se. Instead, they claim that the possible cause for impairment in children with ASD, and the reason for the detected difficulty, lie in the mechanisms for the processing of the temporal structure of events, that is, the ability to ascertain whether the events are ongoing or completed. Finally, Su et al. suggested that the difficulties that Mandarin-speaking children with ASD have in interpreting *wh*-words are located within the domain of semantics rather than intonation. This is because the difficulty was also present in questions versus statements that are not marked by an intonation shift, but depend on the relation of the *wh*-word with the universal quantifier *all*, in other minimal pair sentences (Su et al. 2014). In conclusion, although several studies identified weaknesses in the (morpho)syntax of high-functioning children with autism, even when they were children who scored within the norms on general verbal tasks (Su et al. 2014; Terzi et al. 2014), it is unclear whether these weaknesses result from deficits within (morpho)syntax or from the interface of (morpho)syntax with other domains of language.

The present study follows on the study by Terzi et al. (2014) and addresses this issue. In particular, it poses the question whether the difficulties in the reference and production of clitic pronouns that high-functioning Greek-speaking children with ASD demonstrate result from difficulties with aspects of (morpho)syntax or from difficulties at the interface of (morpho)syntax with pragmatics and/or prosody. English does not have clitic pronouns, hence, the two languages cannot be compared in this area of grammar for possible insights. To be able to establish whether deficits in a (morpho)syntactic phenomenon result from problems in (morpho)syntax per se, or from problems in pragmatics and/or prosody at their interface with (morpho)syntax, it is necessary to test a range of structures including those that implicate (morpho)syntax and those that implicate (morpho)syntax with pragmatics and prosody. To ensure that potential deficits are not the consequence of low non-verbal abilities of the participants, the present study focuses on high-functioning children with ASD, that is, on children who score within norms on general non-verbal tasks.

## Pronominal Clitics: Syntax, Pragmatics, Prosody

Greek, along with several Romance languages (e.g., Italian, Spanish, French), has two forms of object pronouns: Strong and weak, cf. (1) and (2) respectively. The latter are also known as clitics.
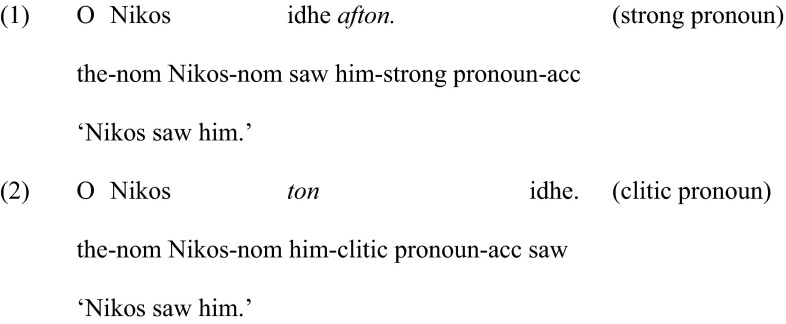


Like all pronouns, clitic pronouns cannot refer to an entity within the sentence in which they occur. Instead, they pick up their reference from a prominent antecedent in the immediately previous linguistic context (i.e., the discourse) (Anagnostopoulou 1999; Mavrogiorgos 2010). Prominent antecedents are those that are most recently introduced or updated, following Heim’s (1982) *Prominence* Condition. The linguistic information we make available will determine which antecedent is prominent in the immediately preceding context, and, as a result, will determine whether or not we will elicit a pronoun or the corresponding noun phrase. Hence, if we ask the question ‘What is the elephant doing to the monkey?’, the felicitous answer will include pronouns ‘He is kicking *it*’, because both *the elephant* and *the monkey* are prominent by being the most recently introduced elements into the linguistic context. On the other hand, if we ask ‘What is the elephant doing?’ the target response will be ‘He is kicking *the monkey*.’ because ‘the monkey’ was not introduced in the previous linguistic context, thus, it is not prominent in the discourse. Example (3) illustrates the equivalent context in Greek, and (4) illustrates the use of a clitic pronoun, *tin*, in the response.
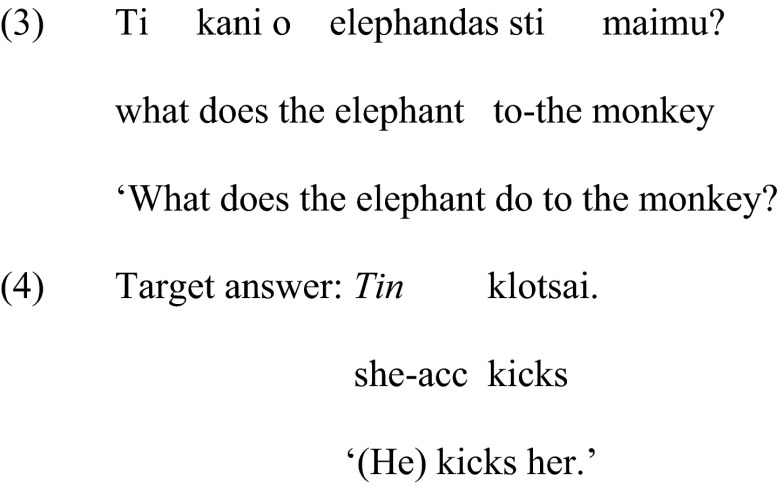


Clitics are encountered systematically also in structures that involve a clitic and the associated definite noun phrase in the same sentence, namely, in the structures known as clitic left dislocation, shown in (5) below.
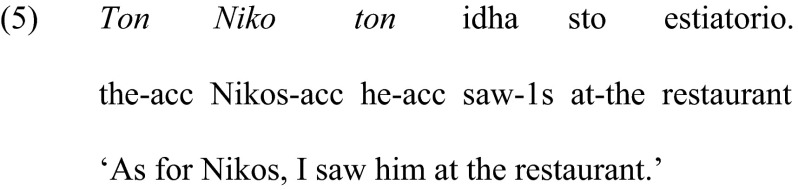


Clitic left dislocation structures are well defined and described by contemporary linguistic theory (Anagnostopoulou 1997; Cinque 1997). They involve a clitic pronoun (*ton* in the sentence above) that is preceded by a noun phrase (*ton Niko*) in the very beginning of the sentence. The clitic and the noun phrase are part of the syntactic construct known as *predicate variable chain*, headed by the clitic (Anagnostopoulou 1997). The presence of the co-referential noun phrase and the predicate variable chain renders clitic left dislocation structures, as in (5), syntactically more complex than structures that involve just a simple clitic, as in (4). The noun phrase in the left of the clitic refers to old or given information for the addressee, either because it occurred in the previous linguistic context, or because it is sufficiently salient in the extralinguistic context (Cinque 1997). This noun phrase is not stressed and there is no pause between the noun phrase and the clitic that follows (Anagnostopoulou 1997). It is generally accepted that there is a relation between the noun phrase and the clitic, something that one can easily perceive intuitively since the two refer to the same individual. Recently it has also been proposed that clitic left dislocation involves explicit or implicit contrasting (Arregi 2003; López 2009). In the example (5) above, the contrast could be ‘As for Nikos, I saw him at the restaurant (and not at the cinema)’.

There is one more structure that involves a fronted noun phrase, the one known as focus structure (Cinque 1997; Rizzi 1997), illustrated in (6). In the focus structure, the fronted noun phrase at the beginning of the sentence bears focal stress, conventionally indicated by upper case letters.
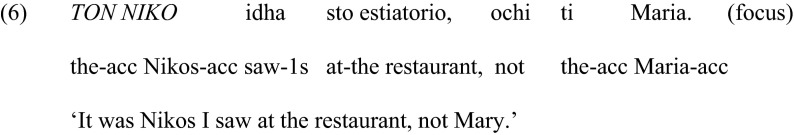


The fronted noun phrase in (6) conveys new information and is explicitly or implicitly contrasted with another individual or object. In the above example the two individuals are explicitly contrasted, that is, we are dealing with an instance of contrastive focus. It is generally assumed that the noun phrase in (6) originates in object position, after the verb, and moves syntactically to the beginning of the sentence. This process renders the structure syntactically complex. Importantly for our study, unlike in (5), a co-referential clitic is not allowed in focus structures, as illustrated in (7) below (Grillia 2008; Rizzi 1997; Tsimpli 1995).
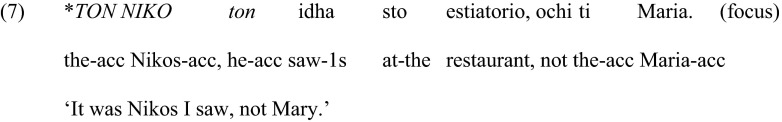


The present study examines the above structures (clitic, clitic left dislocation, focus), in which a clitic may, or may not, occur. In addition, it examines whether individuals with ASD know when not to use a clitic in much simpler structures syntactically, namely, as answers to simple *wh*-questions, in which the answer requires just a noun phrase, and, crucially, does not allow for the corresponding clitic, as in the dialogue in (8)–(9). As we noted earlier, a clitic is not possible in place of the noun phrase ‘ti Maria’, (9), since this noun phrase has not been mentioned in the preceding question, (8).
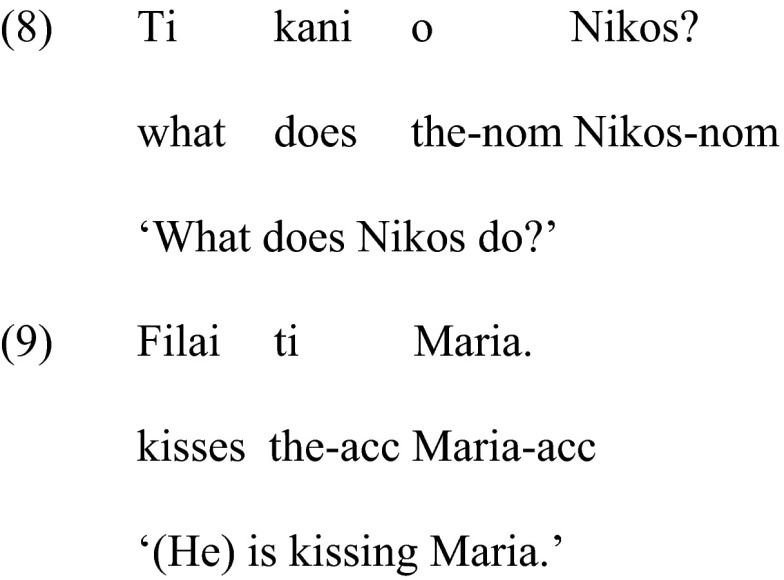


## Aims of the Present Study

The first aim of the study was to replicate the findings of our original study (Terzi et al. 2014) in a new cohort of high-functioning Greek-speaking children with ASD in order to find out whether the new group of children would also demonstrate similar difficulties in the comprehension and production of pronominal object clitics. The second aim was to test whether the difficulties with clitics have a purely (morpho)syntactic source or whether they are the consequence of difficulties at the interface of (morpho)syntax with discourse or with discourse and prosody, given that clitics interact with all three domains. To address these aims, we administered comprehension and production tasks that included environments for clitics and noun phrases (currently referred to as determiner phrases = DPs). The environments for clitics included simple clitics that are felicitous on the basis of the *prominence condition* and clitic left dislocation that requires the use of clitics on the basis of a more complex syntax. The environments for noun phrases included noun phrases that are felicitous on the basis of the discourse and focus structures that require noun phrases on the basis of the discourse and its mapping to specific prosody. If the children’s difficulty with clitics is due to syntactic difficulties, the difference between children with ASD and TD controls should be exacerbated in clitic left dislocation contexts because they are syntactically more complex due to the *predicate variable chain* they implicate. If the children’s difficulty with clitics arises because they do not know that a clitic should refer to a prominent entity in the preceding discourse or they cannot tell what the prominent entity is in the discourse, then we would expect them to sometimes use noun phrases instead of clitics. If the children have difficulties at the interface of (morpho)syntax with discourse, in the sense that they cannot make use of discourse cues showing that the referent is old/new or they cannot tell which referent is salient in order to use the felicitous (morpho)syntactic structure (clitic or noun phrase), they should make errors not only in the use of clitics, but also in the use of noun phrases. This predicts the use of noun phrases instead of clitics when the referent has already been mentioned in the discourse and the use of clitics instead of noun phrases when the referent is new. Finally, if the children have difficulty at the interface of (morpho)syntax with discourse and prosody, that is, they cannot make use of prosodic cues in order to use the felicitous linguistic expression (noun phrase, in this instance), they should use clitics instead of noun phrases in focus structures.

## Method

### Participants

Twenty high-functioning children with ASD participated in the study and twenty typically developing controls, matched on their age and language abilities on the basis of the Greek version of the Peabody Picture Vocabulary Test (PPVT) (Simos et al. 2011), see Table 1 for the children’s characteristics.

**Table 1:** Results from baseline tasks

	ASD children (N = 20)	TD children (N = 20)	*p* value
Raven’s standard score			
Mean	104.8	95.5	<0.05
Range	80–130	80–115	
SD	18.2	7.9	
PPVT raw score			
Mean	92.9	93.1	>0.1
Range	76–123	74–122	
SD	14.9	14.7	
DVIQ raw score			
Mean	20.8	21.4	>0.1
Range	15–24	17–24	
SD	2.3	2.1	
Listening span raw score			
Mean	4.6	4.8	>0.1
Range	0–12	0–11	
SD	4.06	4.02	
Listening span span			
Mean	0.75	0.8	>0.1
Range	0–2	0–2	
SD	0.72	0.77	
Digit span raw score			
Mean	7.9	8.4	>0.1
Range	0–24	5–17	
SD	5.9	3.5	
Digit span span			
Mean	2.2	2.4	>0.1
Range	0–5	2–4	
SD	1.23	0.59	

The Raven’s scores are from Raven’s Coloured Matrices test (Raven 1998), the PPVT scores are from the Greek version of the Peabody Picture Vocabulary Test (PPVT) (Simos et al. 2011), the DVIQ scores are from the Diagnostic Test of Verbal Intelligence (DVIQ) (Stavrakaki and Tsimpli 2000), the listening span and the digit span scores are from the adapted versions of the working memory battery (Pickering and Gathercole 2001) for Greek (Chrysochoou et al. 2013)

The children with ASD had a mean age of 6;11 (SD in months: 13.9; range in months 65–104) and the TD children a mean age of 6;7 (SD in months: 11.5; range in months 61–98), *F*(1, 39) = 1.350, *p* = 0.25, η_p_^2^ = 0.034. The children with ASD were matched individually to TD children on the raw score of the PPVT by ±5 points difference. The children with ASD were attending private clinics in Athens and Patras specialized in children with ASD, and were holding a community diagnosis of a Pervasive Developmental Disorder (PDD) according to DSM-IV-TR criteria (American Psychiatric Association 2000). Twelve were children with Autistic disorder, six with Asperger and two with PDD-NOS. None of the children had a diagnosis of CDD/Rett. The children were referred to us and the child psychiatrist of our team (KF), an ADOS trainer, corroborated the diagnosis with the use of Autism Diagnostic Observation Schedule, Second Edition—ADOS-2 (Lord et al. 2012). The children were included if their scores met at least the cutoff scores for ASD. Due to the small number of participants and the changes in the concept in DSM-5 (American Psychiatric Association 2013), we chose not to retain the specific diagnostic subcategories of DSM-IV-TR and all cases were included as an ASD group, given that they are not distinguished by DSM-5. Moreover, based on the chart review, they all met the DSM-5 criteria for an ASD diagnosis. The typically developing children were recruited from public schools of Patras. Teachers were asked to identify children with a known or suspected developmental disorder, and these children were excluded. None of the children in the typically developing group had a history of speech or language delay or disorders and no concerns about their development were expressed by their parents and teachers. The data of both groups were collected by a certified speech-language pathologist research assistant with experience in children with developmental disorders, who could easily detect whether the TD children were indeed typically developing. Ethical approval for the study was provided by the Research Ethics Committee of the Ministry of Education (Institute of Educational Policy). All parents provided informed written consent for their children’s participation.

### Measures

Children were administered a battery of baseline tests to ascertain their verbal, non-verbal, and memory abilities. The children’s non-verbal abilities were assessed via the Raven’s Coloured Matrices test (Raven 1998). Their grammatical abilities were measured via the (morpho)syntax subtest of the Diagnostic Test of Verbal Intelligence—DVIQ (Stavrakaki and Tsimpli 2000). The Greek version of the PPVT (Simos et al. 2011) assessed the children’s vocabulary abilities and was used for matching of the two groups. The children’s working memory was assessed using a listening span test (Pickering and Gathercole 2001) and a backwards digit span test (Pickering and Gathercole 2001), adapted for Greek (Chrysochoou et al. 2013).

Table 1 shows the children’s performance on the baseline tasks. All children had a standard score of 80 or above on the Raven’s Coloured Matrices and, thus, were characterized as high-functioning. They all scored above 80 on the PPVT, which indicates that they also had language abilities within the norms. The children with ASD had slightly higher scores on the non-verbal abilities compared to the TD children, *F*(1, 39) = 4.324, *p* = 0.044, η_p_^2^ = 0.102,^1^ but there was no significant difference between the two groups on their grammatical abilities, as measured through the DVIQ, *F*(1, 39) = 0.87, *p* = 0.357, η_p_^2^ = 0.022, their vocabulary abilities, as measured through the PPVT, *F*(1, 39) = 0.003, *p* = 0.958, η_p_^2^ < 0.001, and their working memory, as measured through the listening span and the backwards digit span tests, listening span raw score: *F*(1, 39) = 0.025, *p* = 0.876, η_p_^2^ = 0.001; listening span: *F*(1, 39) = 0.045, *p* = 0.833, η_p_^2^ = 0.001; backwards digit span raw score: *F*(1, 39) = 0.086, *p* = 0.771, η_p_^2^ = 0.002; backwards digit span: *F*(1, 39) = 0.433, *p* = 0.514, η_p_^2^ = 0.011.

The children’s comprehension and production of clitics and noun phrases was measured in a number of environments using a comprehension and a production task.

#### Comprehension Task

A picture selection task was designed to assess the comprehension of the reference of clitics in two conditions: (1) As simple clitics, and (2) in clitic left dislocation structures. Six sentences were created for each condition. Each sentence was presented together with three pictures; one was the target picture and the other two were foils. The sentences were pre-recorded by two female native speakers of Greek using normal speed and natural intonation in a noise isolated booth to ensure that all children heard the sentences pronounced in exactly the same manner. Adobe Audition was used to edit the recorded sentences. The pictures were created by a professional designer and care was taken to avoid biases due to the size and prominence of the figures. We describe the material below for each condition and present representative sets of sentences and pictures.

#### Condition 1: Clitics

To test the comprehension of clitics we used the items from Terzi et al. (2014). The sentences were created using six actional verbs (*pleno* ‘wash’, *luzo* ‘shampoo’, *dino* ‘dress’, *skupizo* ‘wipe’, *skepazo* ‘cover’, *haidevo* ‘caress’). The subject of each sentence was a proper name or a kinship term and the clitic was always the object of the sentence, as shown in (10) below.
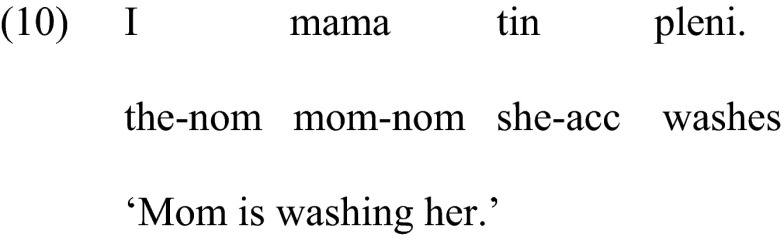


**Fig. 1:**
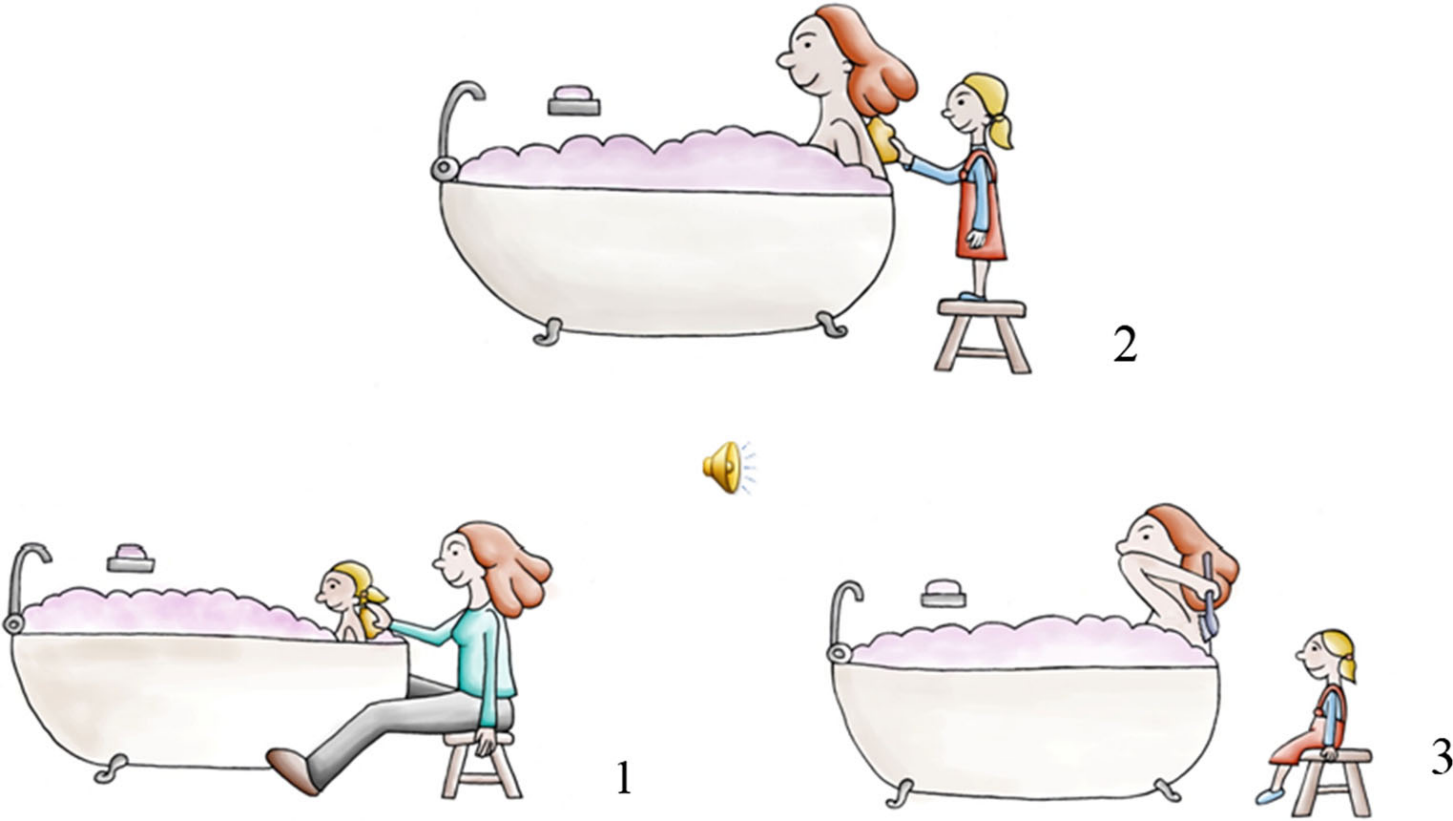
Sample of pictures used for the comprehension of clitics/clitic left dislocation

To avoid gender cues, both the subject and the object had the same gender, masculine or feminine. Figure 1 illustrates the slide with the pictures presented with this sentence.

The target picture of the slide showed mom washing Mary, (Picture 1). The second picture showed the same persons with the thematic roles reversed, i.e., Mary washing mom, (theta-role reversal), (Picture 2), and the third picture depicted the person mentioned in the sentence, i.e., mom, doing a reflexive action, that is, washing herself (reflexive interpretation), while Mary was watching nearby, (Picture 3). The position of the three pictures in each slide was pseudo-randomized to ensure that the correct picture was not presented in the same position. At the beginning of the testing participants were presented with a picture that had all characters of a family and their names. This ‘family’ picture was kept next to the scene during testing to avoid errors because children could not remember the names of the characters. The names of the characters were also repeated each time a new slide was presented. Comprehension of pronominal clitics or pronouns in general, assessed via such tasks, essentially amounts to assessing the knowledge of picking the right referent of a pronoun (Chien and Wexler 1990).

#### Condition 2: Clitic Left Dislocation

The same six actional verbs were also used in this condition. The subject of each sentence was null this time and the clitic and associated noun phrase were the object of the sentence, as shown in (11) below. We chose a sentence with a null subject so that it is minimally different superficially from the previous sentence that tested comprehension of simple clitics in the clitics condition.
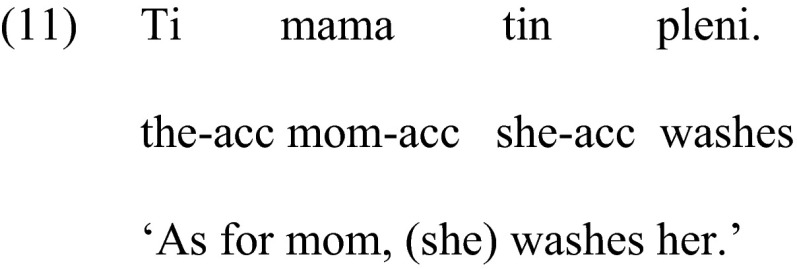


The null subject corresponded to a character in the picture that had the same gender as the object of the sentence, that is, masculine or feminine, in order to avoid gender cues. The pictures were the same as in Condition 1, illustrated in Fig. 1. For the sentence in (11) the target picture showed a female character, e.g., Mary, washing mom, (Picture 2). The second picture showed the reversed action, mom washing Mary (Picture 1). The third picture depicted the person mentioned in the sentence, i.e., mom, doing a reflexive action, that is, washing herself, (reflexive interpretation), while Mary was watching nearby, (Picture 3). The position of the three pictures in each slide was pseudo-randomized and all sentences were presented in pseudorandom order.

#### Production Task

An elicitation task with five different conditions was used to elicit the production of: (1) Simple clitics, (2) clitic left dislocation structures, (3) simple noun phrases that were present in the introductory sentences (DP1), (4) simple noun phrases that were not present in the introductory sentences, (DP2), and (5) noun phrases in focus structures. Pictures and introductory sentences were used to create the appropriate context for the use of the five structures. The pictures were created by a professional designer and care was taken to avoid biases due to the size and prominence of the figures. Each condition was presented in a block and consisted of six sentences, hence, the task elicited 30 sentences. The blocks were presented in the order: Clitic, clitic left dislocation, DP1, focus, DP2, that is, first the two blocks involving clitics and then the three blocks involving DPs. This ensured that a carry over effect could be attested only from the clitic left dislocation condition to the DP1 condition. All verbs were actional transitive verbs that cannot surface without their direct object (*filao* ‘kiss’, *klotsao* ‘kick’, *agaliazo* ‘hug’, *dagono* ‘bite’, *tsimbao* ‘pinch’) and all arguments of the verbs were animals (*arkuda* ‘bear’, *gata* ‘cat’, *elafi* ‘deer’, *elefandas* ‘elephant’, *katsika* ‘goat’, *liondari* ‘lion’, *maimu* ‘monkey’, *lagos* ‘rabbit’, *provato* ‘sheep’, *likos* ‘wolf’). Below we describe the material for each condition and present representative sets of sentences and pictures.

#### Condition 1: Clitics

**Fig. 2:**
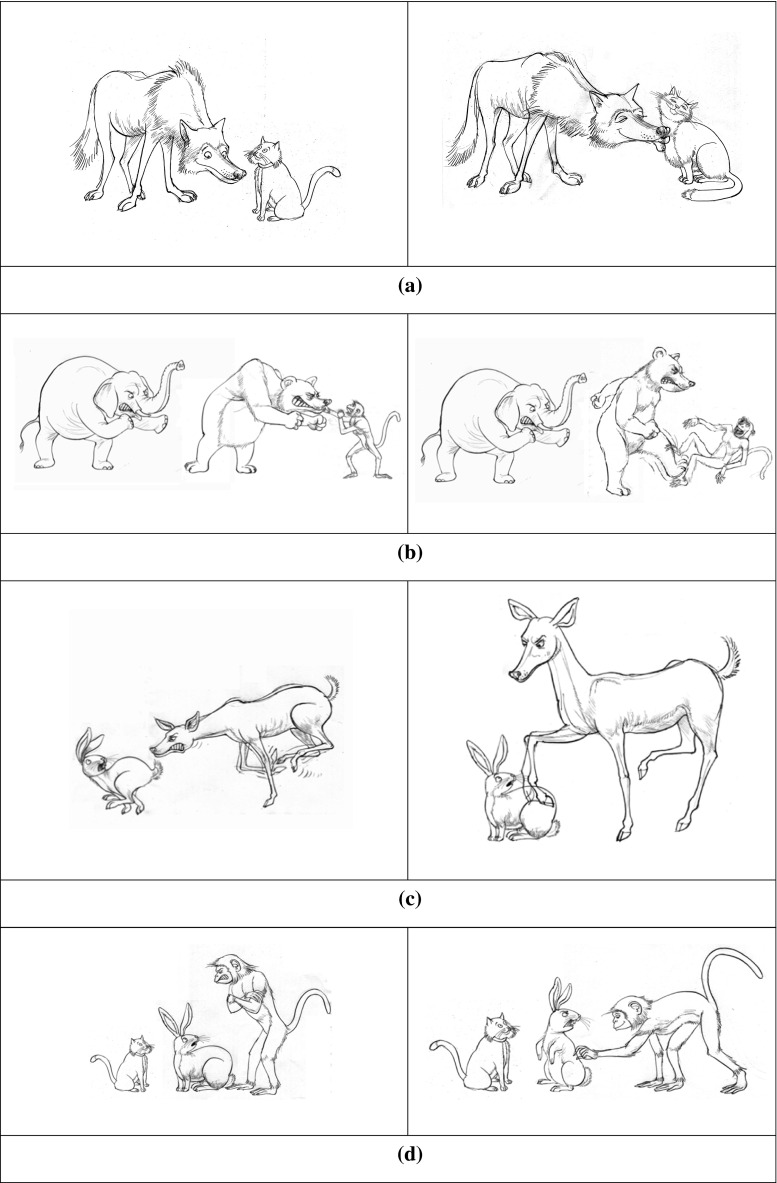
Sample of pictures used for the elicitation task. **a** Elicitation of clitics. **b** Elicitation of clitic left dislocation. **c** Elicitation of noun phrases (with/without introduction of characters). **d** Elicitation of noun phrases in focus structures

To elicit clitics we used the elicitation task of Chondrogianni et al. (2015). Children were shown two pictures with two characters each on a computer screen, as in Fig. 2a.

Children were introduced to the characters in the first picture and while they were shown the second picture they were asked what character A did to character B, as in (12). The response should elicit a clitic pronoun, as in (13).
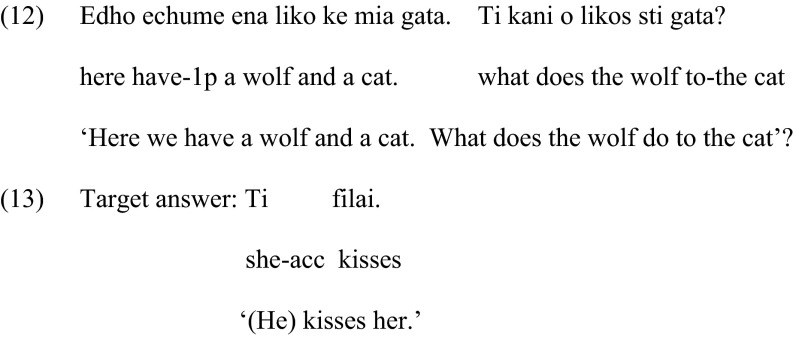


#### Condition 2: Clitic Left Dislocation

As in the previous condition, two pictures were shown to the children, and a question was asked. However, in order to create a felicitous context for clitic left dislocation, each picture contained three animals, as shown in Fig. 2b. A picture with three animal characters is also able to accommodate the implicit contrasting that, for some researchers, can be present in clitic left dislocation, and, importantly, it matches the pictures used to elicit the focus structure. This was a sentence completion task; the experimenter provided the first noun phrase of the answer, as shown in (14), and the children had to complete the sentence, as shown in (15). As previously, the first picture was used to introduce the characters, while the second was used together with the question in order to elicit the clitic left dislocation structure.
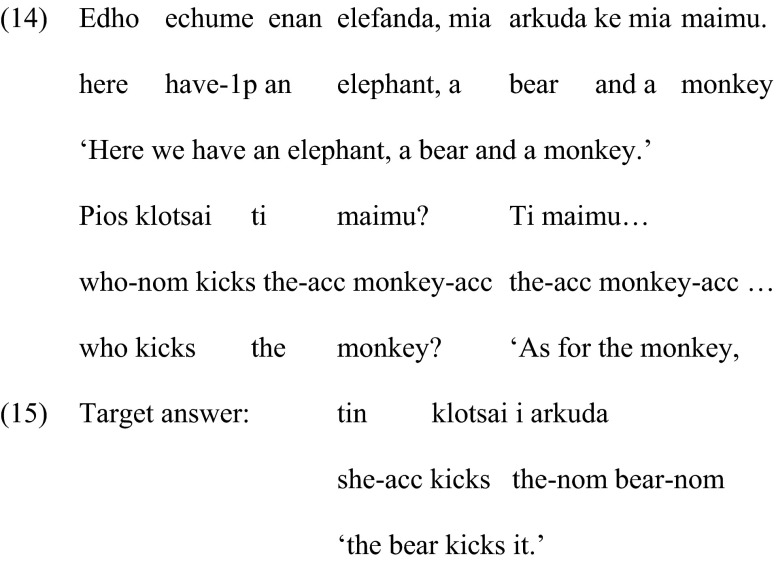


#### Condition 3: DP1—Noun Phrase Present in the Introductory Sentences

In this condition we tested whether children were able to use an object noun phrase when the characters were present in the introductory sentences, but they were not contained in the immediately preceding context, that is, in the eliciting question. Children saw two pictures, with two characters each, as in the condition with clitics, see Fig. 2c.

The context preceding the question requesting a noun phrase was the same as in the condition with clitics, namely, it introduced the characters in the picture. However, the eliciting question did not mention the object noun phrase, but the subject and a proform of the verb, i.e., *do*, as shown in (16). This is why the target response, (17), is an object noun phrase and not a clitic.
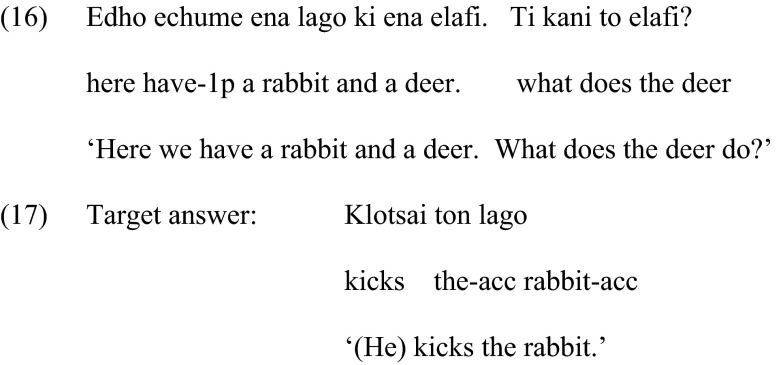


#### Condition 4: DP2—Noun Phrases Not Present in the Introductory Sentences

This condition is almost identical to Condition 3. The only difference is that the characters were not present at the beginning of each trial, thus providing a stronger environment for a noun phrase in the response. Therefore, this condition tested whether children are sensitive to the discourse in terms of using a noun phrase for characters that are new to the speaker not only because they were not present in the question eliciting the noun phrase response, but also because they were not present anywhere in the preceding linguistic context. The eliciting question was exactly the same as in Condition 3. Therefore, comparison between Condition 3 and 4 can demonstrate whether and how children are sensitive to discourse information. Example (18) illustrates the prompt and (19) the target response.
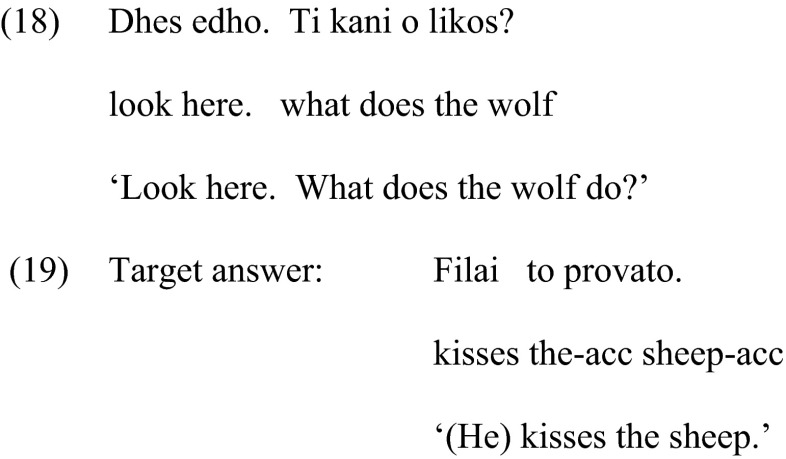


#### Condition 5: Noun Phrase in a Focus Structure

This condition tested the children’s knowledge that a direct object clitic *cannot* be used in sentences in which the associated direct object noun is focused in sentence initial position. Similarly to the clitic left dislocation, this was a sentence completion task with two pictures, each one of which contained three animal characters, as shown in Fig. 2d.

The interviewer asked a question such as in (20), and then started answering it by producing the first noun phrase with focus intonation. The three animals in the picture made the contrastive focus interpretation pragmatically appropriate for the response in (21).
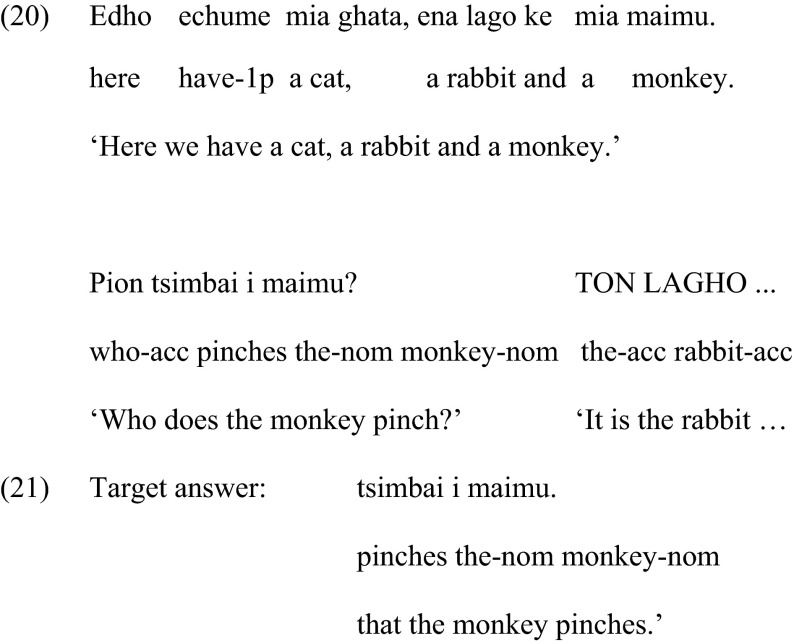


### Procedure

Each child was seen individually on 2 or 3 occasions, depending on their attention. The children with ASD were seen in the clinic whereas the TD children were seen in their school.

## Results

**Fig. 3:**
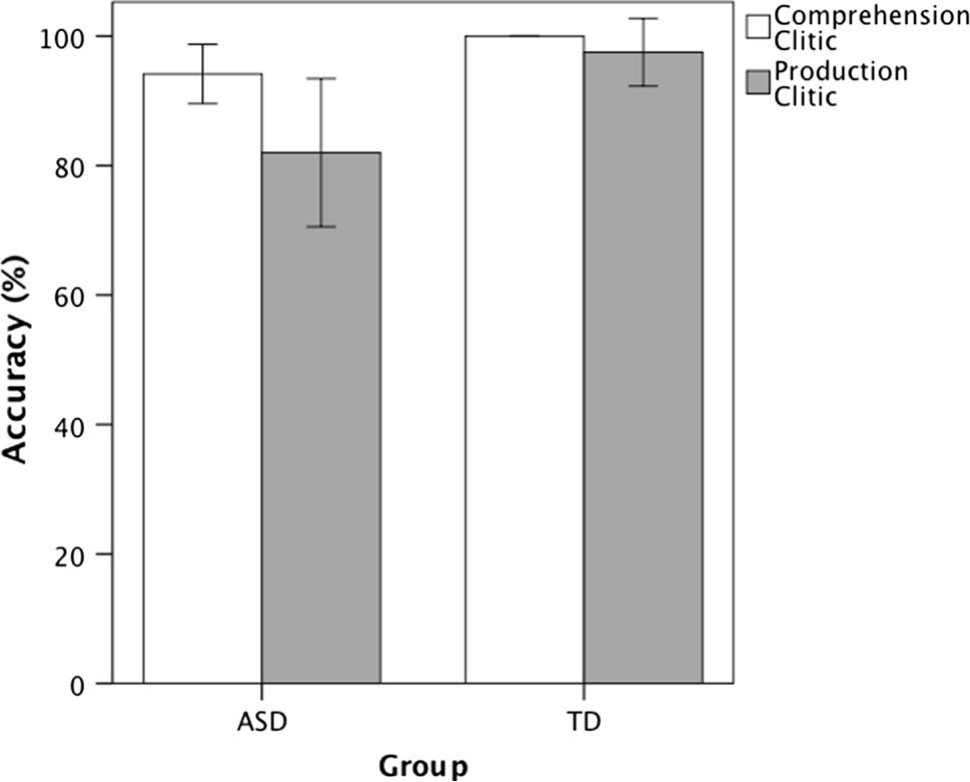
Mean difference in the comprehension and production accuracy of clitics in children with Autism Spectrum Disorder (ASD) compared to typically developing (TD) children. The children with ASD had lower accuracy than the TD children and overall production scores were lower than comprehension scores. Standard errors are represented in the figure by the *error bars* attached to each column

The first analysis tests whether or not the cohort of children with ASD in the present study perform in a similar manner as the children with ASD in Terzi et al. (2014). Figure 3 shows the accuracy in the comprehension and production of clitics in the children with ASD and the TD controls. A repeated measures ANOVA with Group as a between subjects factor and Task as the within subjects factor revealed a significant main effect of Group, *F*(1, 38) = 10.432, *p* = 0.003, η_p_^2^ = 0.215, a significant main effect of Task, *F*(1, 38) = 5.617, *p* = 0.023, η_p_^2^ = 0.129, and no significant interaction between Group and Task, *F*(1, 38) = 2.440, *p* = 0.127, η_p_^2^ = 0.06. This indicates that overall the children with ASD (*M* = 88.1 %) had lower accuracy than the TD children (*M* = 98.8 %) and accuracy in the comprehension task (*M* = 97.1 %) was higher than in the production task (*M* = 89.7 %). This replicates the findings of the study by Terzi et al. (2014).

The error analysis in the comprehension task showed that the small number of errors in the children with ASD (7 out of 7) consisted of selecting the picture with reversed thematic roles. In terms of the production task, most errors in the children with ASD (14 out of 21) and all errors (3 out of 3) in the TD children consisted in using a noun phrase instead of a clitic (The wolf is kissing the cat) whereas the remaining 7 errors in the children with ASD were errors of omission (The wolf is kissing).

**Fig. 4:**
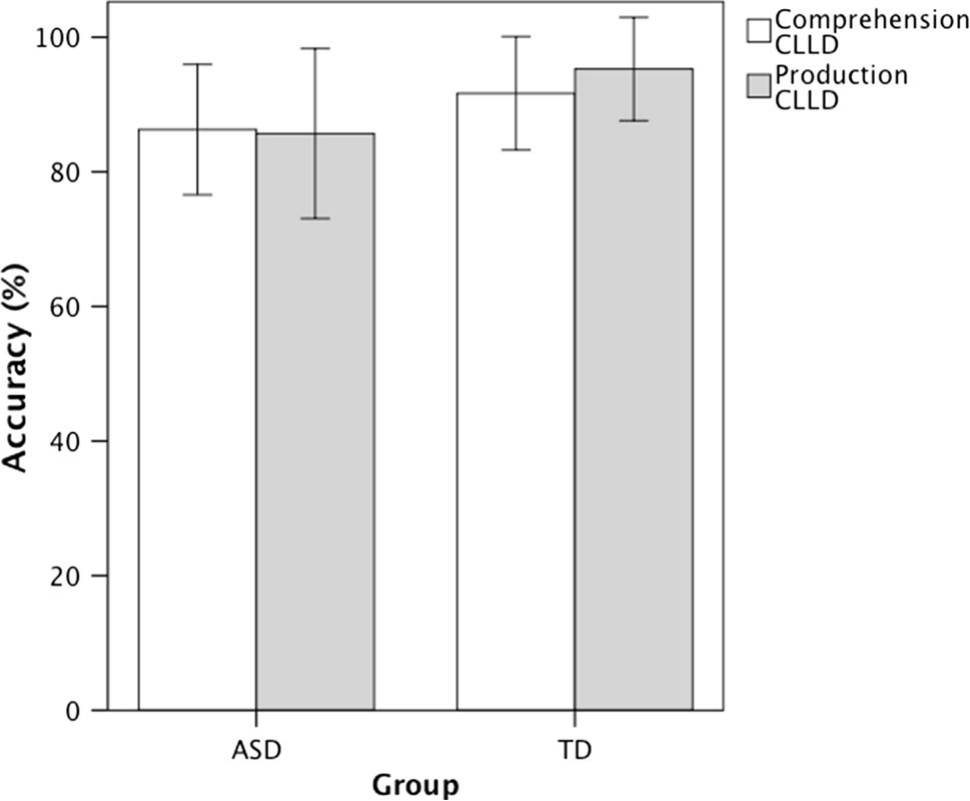
Accuracy in the comprehension and production of clitic left dislocation (CLLD) in children with Autism Spectrum Disorder (ASD) compared to typically developing (TD) children. There was no between group difference and no difference between comprehension and production. Standard errors are represented in the figure by the *error bars* attached to each column

The next analysis tests whether an increase in syntactic complexity will lead to an even lower accuracy in the comprehension and production of clitics in children with ASD by investigating the comprehension and production of clitic left dislocation structures in which the noun phrase and the clitic are co-referential and involve a predicate chain. Figure 4 shows the accuracy in the comprehension and production of clitic left dislocation. A repeated measures ANOVA with Group as a between subjects factor and Task as the within subjects factor revealed no significant main effects of Group, *F*(1, 27) = 2.602, *p* = 0.118, η_p_^2^ = 0.088, Task, *F*(1, 27) = 0.081, *p* = 0.779, η_p_^2^ = 0.003, and no significant interaction between Group and Task, *F*(1, 27) = 157, *p* = 0.695, η_p_^2^ = 0.006, indicating that the children with ASD were as accurate as the TD in clitic left dislocation and there was comparable performance in the comprehension and production tasks.

The error analysis in the comprehension task showed that most errors in the children with ASD (12 out of 15) consisted of choosing the picture with the reversed thematic roles, whereas the remaining 3 errors consisted of choosing the distracter picture. Similar results were obtained in the error analysis of the production task. Most errors in the children with ASD (6 out of 7) and all errors (3 out of 3) in the TD children consisted of reversal of thematic roles (The monkey … kicks the bear) whereas the remaining 1 error in the children with ASD was an error of omission.

**Fig. 5:**
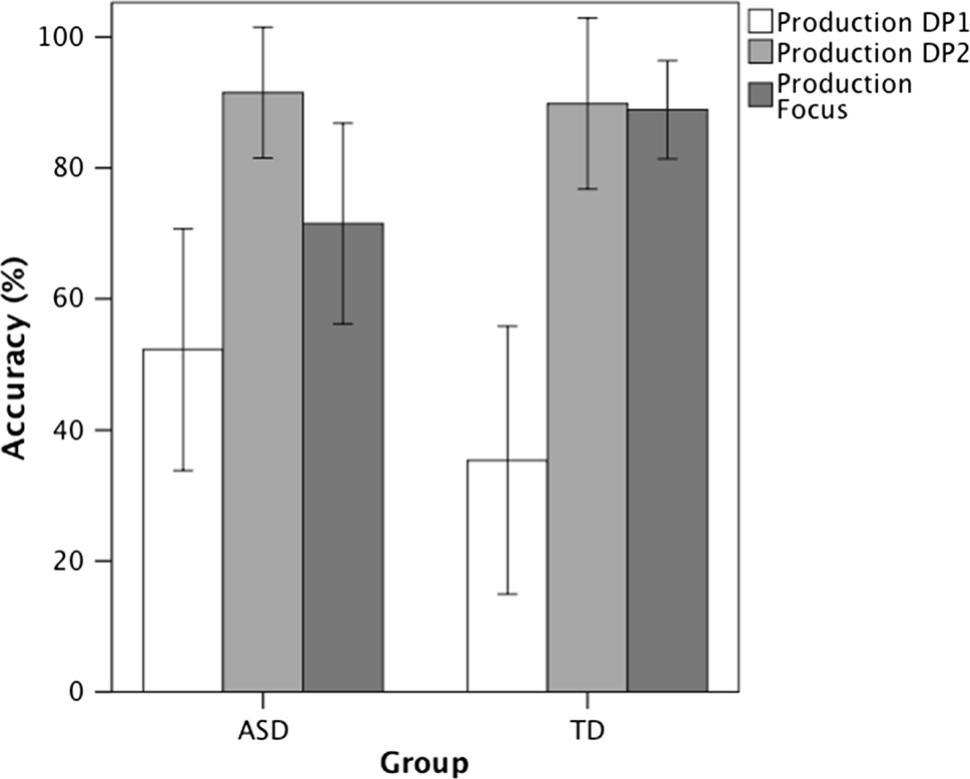
Accuracy in the production of simple noun phrases presented with an introductory sentence (DP1), simple noun phrases presented without an introductory sentence (DP2), and focus sentences (Focus) in children with Autism Spectrum Disorder (ASD) compared to typically developing (TD) children. The children with ASD were less accurate than the TD children in focus sentences. Standard errors are represented in the figure by the *error bars* attached to each column

The third analysis investigates three contexts, in which noun phrases rather than clitics are required and tests whether children with ASD are sensitive to the discourse (old or new information, prominence) and prosody cues for the use of noun phrases. Figure 5 shows the accuracy in the production of DP1, DP2, and DP in focus. A repeated measures ANOVA with Group as a between subjects factor and noun phrase type as the within subjects factor revealed no significant main effect of Group, *F*(1, 36) = 0.004, *p* < 0.949, η_p_^2^ < 0.001, a significant main effect of noun phrase type, *F*(2, 72) = 25.807, *p* < 0.001, η_p_^2^ = 0.418, and a significant interaction between Group and noun phrase type, *F*(2, 72) = 3.148, *p* = 0.049, η_p_^2^ = 0.080, indicating that the two groups of children performed differently in the three conditions.

Comparisons between the three noun phrase types in each group separately and between group comparisons for each noun phrase type separately were conducted to uncover the source of this interaction. The within group analyses showed that in the ASD group there was a significant main effect of noun phrases type, *F*(2, 18) = 9.932, *p* = 0.001, η_p_^2^ < 0.525, due to a significant difference between DP1 (*M* = 52.3 %) and DP2 (*M* = 91.5 %) (*p* = 0.001), but no significant differences between DP1 and focus (*M* = 71.5 %) (*p* = 0.32) or DP2 and focus (*p* = 0.15). In the TD children there was also a significant main effect of noun phrases type, *F*(2, 16) = 15.591, *p* < 0.001, η_p_^2^ < 0.661, due to significant differences between DP1 (*M* = 35.4 %) and DP2 (*M* = 89.8 %) (*p* < 0.001) and between DP1 and focus (*M* = 88.9 %) (*p* < 0.001), but no significant difference between DP2 and focus (*p* = 1). The between group analyses showed no significant differences between the groups in DP1 (*F*(1, 38) = 1.628, *p* = 0.21, η_p_^2^ = 0.041) and DP2 (*F*(1, 38) = 0.008, *p* = 0.929, η_p_^2^ < 0.001), but the children with ASD had a significantly lower accuracy than the TD in the focus condition (*F*(1, 38) = 4.252, *p* = 0.046, η_p_^2^ = 0.106.

The error analysis showed that in the DP1 condition the most frequent error was the production of clitics (ASD: 50 out of 56 errors; TD: 75 out of 76 errors). The children with ASD showed also 4 errors of omission and 2 errors of reversal and the TD children showed 1 error of omission. By ‘reversal’ we refer to the responses in which children reversed the thematic roles of the target sentence. In the DP2 condition, the children with ASD showed an equal number of errors in inappropriate use of clitics (3 errors), omissions (4 errors) and reversals (3 errors) and the TD children showed 8 errors of inappropriate use of clitics and 3 errors of omission. In the focus condition, the largest number of errors in the children with ASD involved inappropriate use of clitics (15 errors) and lack of sensitivity to the context (10 errors), and a small number of errors (3 errors) involved reversal. The TD children showed an equal number of inappropriate use of clitics (4 errors), lack of sensitivity of context (2 errors) and reversals (3 errors). By ‘lack of sensitivity to the context’ we refer to responses that were correct in terms of who does what to whom, but the answer was not appropriate for the focus context, since the beginning of the answer was provided by the experimenter. These were responses of the type: Agent Verb Patient, i.e., *the monkey pinches the rabbit* in the case of (20)-(21).

## Discussion

The study reported here aimed at shedding light as to whether the difficulties that children with ASD have in the comprehension and production of clitics, (Terzi et al. 2014), are caused by difficulties within the domain of (morpho)syntax, at the interface of (morpho)syntax with discourse/pragmatics, or at the interface of (morpho)syntax with discourse and prosody. The first objective was to replicate the study by Terzi et al. (2014) in a new group of high-functioning Greek-speaking children of similar age with ASD. Provided this was accomplished, the second objective was to investigate whether the difficulties with clitic pronouns have a purely (morpho)syntactic source, or whether they are the consequence of difficulties at the interface of (morpho)syntax with discourse or with discourse and prosody, given that clitics interact with all three.

To address these objectives, we administered comprehension and production tasks that included environments for clitics and noun phrases. The environments for clitics assessed simple clitics that are felicitous on the basis of the prominence of their referent in the discourse, and clitic left dislocation that requires the use of clitics on the basis of discourse, but with more complex syntax. The environments for noun phrases included noun phrases that are felicitous on the basis of the discourse, and focus structures, which require noun phrases on the basis of discourse and prosody. If the ASD children’s difficulty with clitics is due to syntax, the difference between children with ASD and TD controls should be exacerbated in clitic left dislocation contexts. If the children’s difficulty is due to not knowing that a clitic should be used to refer to a prominent entity in the preceding discourse, or that they cannot tell what the prominent entity in the discourse is, they then should sometimes use noun phrases instead of clitics. If their difficulty is at the interface of (morpho)syntax with discourse, that is, they cannot make use of discourse cues that show that the referent is old/new (clitic or noun phrases respectively), they should make errors not only in the use of clitics, but also in the use of noun phrases. This predicts the use of noun phrases instead of clitics when the referent is old and/or prominent and the use of clitics instead of noun phrases when the referent is new. If the children’s difficulty reflects difficulties at the interface of (morpho)syntax with discourse and prosody, that is, children with ASD cannot make use of prosodic cues in order to use the felicitous linguistic expression, they may use clitics instead of noun phrases in focus structures. Finally, if the children have no grasp of any of the above requirements for the use of clitics and noun phrases, the result would be chance performance.

The simple clitics results obtained in the current study replicated the findings of Terzi et al. (2014).  Hence, Greek-speaking high-functioning children with ASD fell behind their language matched controls on both the comprehension and the production of object clitics, with the gap being wider for production. In comprehension, the children with ASD committed the same errors as in the aforementioned study, namely, instead of the target picture, they chose the one in which the characters were reversed. Our comprehension data do not show whether or not the errors are due to difficulties in (morpho)syntax or the interface of (morpho)syntax with discourse or discourse and prosody because the task was not designed to distinguish between these three options. This issue was addressed through the production task however. In the production task, the predominant error was the use of noun phrases instead of clitics, indicating that the children with ASD either do not know that a clitic should be used to refer to a prominent entity in the preceding discourse, i.e., they don’t know *prominence condition* (Heim 1982), or that they cannot tell what the prominent entity is in the discourse. In either case, these errors suggest that their problem lie at the level of discourse, hence, at the (morpho)syntax-pragmatics interface.

The children’s performance in clitic left dislocation, the condition that requires the use of clitics in a syntactically more complex structure than that of simple clitics, showed that the difference between the two groups was not exacerbated, as should be the case if the source of the difficulties was in syntax. In contrast, the children with ASD did not differ from the TD children either in comprehension or in production of clitics in clitic left dislocation environments, suggesting that their problem is not syntactic. Interestingly, both groups of children showed a slightly lower performance in clitic left dislocation structures compared to the condition with simple clitics. This could be a consequence of the fact that clitic left dislocation is a more complex structure than a structure with just a clitic, at least in the sense of involving a chain that consists of the noun phrase and the associated clitic (Anagnostopoulou 1997; Cinque 1997).

Turning now to the structures that elicit a noun phrase, either as a simple answer to a question or as part of a focus structure, and, importantly, do not allow for the presence of a clitic, we found that: (a) The two groups did not differ in the elicitation of simple noun phrases, (b) both groups had lower performance on the first condition (DP1) compared to the second condition (DP2), and (c) the children with ASD performed less well in the elicitation of noun phrases in focus structures compared to their TD controls. We will discuss these three results in turn.

In both the DP1 and DP2 conditions, the predominant error consisted in producing a clitic, rather than a noun phrase. This response constitutes an error because the question eliciting it did not contain the target noun phrase, which would have acted as the prominent element in the immediate discourse and would have triggered the use of a clitic. Both groups seem to consider as relevant discourse information not only the eliciting question, but also what precedes it, namely, the sentence that introduces the characters. As a result, in the condition where the characters were introduced (DP1), there were many more instances of (erroneous) productions of clitics than in the condition where the characters were not introduced (DP2). The similarity between the two groups, together with the fact that both groups were sensitive to the sentence introducing the characters, also indicates that children with ASD have a grasp of the discourse conditions that are relevant for the use of a noun phrase at the exclusion of a clitic.

Apart from the simple clitics, the focus structure was the only other condition in which the two groups differed significantly from each other, with the ASD children performing lower than the TD controls. In this condition, children had to complete a sentence that started with a focused direct object noun phrase, bearing a special focus intonation which is incompatible with a clitic (Cinque 1997; Rizzi 1997). The predominant error of the children with ASD was to produce a clitic, that is, they produced a clitic left dislocation structure. This finding can be interpreted in two ways. The children with ASD could either be insensitive to the intonation pattern or they do not associate this intonation pattern with the particular interpretation that excludes the presence of a clitic. The results from our task cannot differentiate between these two possibilities. However, previous research has demonstrated that high-functioning children with ASD can use prosodic information to disambiguate syntactic structure (Diehl et al. 2015; Su et al. 2014). Therefore, it is most likely that the high-functioning children with ASD of our study are sensitive to the intonation patters of a focus structure, but they simply did not know that it is used to mark a particular interpretation which is not compatible with clitics. Instead, they treat the focused noun phrases in the beginning of the sentence as old information or as the prominent noun phrases and produce a clitic to associate it with them, just as they do in a clitic left dislocation structure. One could think that the partial responsibility for this outcome is the format of the experiment, which introduces the characters before each eliciting question. We already saw from the DP1 versus the DP2 conditions that children were influenced by the background that preceded the eliciting questions. Unlike in the DP conditions, however, the two groups differed in this one. Given that the background information influences similarly the two groups, as concluded from the DP conditions, we are led to conclude that what is responsible for the difference is what follows, namely, the eliciting question with the beginning of the target answer. In particular, we conclude that what the children with ASD do not grasp is that a certain intonation, that of a focused noun phrase, is incompatible with a clitic that refers to it.

To conclude, the systematic investigation of the use of clitics and the corresponding noun phrases has produced two novel findings on the language abilities of children with ASD. High-functioning children with ASD perform less well than TD children only in two of the conditions tested: (1) when they are asked to produce a simple pronominal direct object clitic, and 2) when they have to produce a noun phrase in a focus structure. Their errors in the first context suggest that they have difficulties to identify the prominent item in the discourse, whereas their errors in the second context suggest difficulties to associate a particular intonation with a particular discourse interpretation that excludes clitics. Although independent research is needed to discover how well children with ASD do in identifying what is prominent in the discourse, and how well they do in distinguishing between different intonation patterns outside the domain of clitics, the present findings, coupled with the lack of difference between the two groups in the contexts with increased syntactic complexity (clitic left dislocation), suggest that what looks like a (morpho)syntactic problem is not (morpho)syntactic, but lies at the interface of (morpho)syntax with pragmatics and prosody.

These findings are in line with the studies showing that young Mandarin-speaking high-functioning children with ASD have difficulties to interpret sentences with *wh*-words as statements (Su et al. 2014) and to produce perfective aspect (Zhou et al. 2015), but these difficulties are due to factors outside of syntax proper. The studies showing syntactic deficits that cannot be attributed to some other domain of language are the studies by Perovic et al. (2013a, b), but the participants of these studies were language impaired children, the majority of whom had non-verbal abilities below the norms. The participants of Roberts et al. (2004) who performed low on tense marking were also language impaired and the majority of them had non-verbal abilities below the norms. This suggests that syntax proper may be affected only among such individuals with ASD, whereas the difficulties attested in high-functioning individuals have their source at the interface of (morpho)syntax with other domains of language. Alternatively, such subtle difficulties in high-functioning children with ASD may be residual difficulties that are developmental in nature and may disappear with age. Further research is required to address how low-functioning and/or language impaired Greek-speaking children with ASD perform in the tasks presented in this study and also whether languages with similar types of clitics, notably many Romance languages, show a similar pattern of performance as our study. Finally, a systematic cross-linguistic investigation is urgently needed to address whether there is a common ground in the subtle deficits attested in the (morpho)syntax of high-functioning children with ASD, especially when the structures demonstrating these deficits interface (morpho)syntax with one or more other domains of language (e.g., pragmatics and prosody).

## Footnotes

1 The higher scores on the non-verbal abilities were caused by a pair of ASD-TD children, who had a difference of 35 points on their scores on the Raven’s Coloured Matrices, with the ASD child having a score of 130. By excluding this pair from the analyses, the difference between the children with ASD and the TD children on their non-verbal abilities disappears, *F*(1, 38) = 3.113, *p* = 0.086, η_p_^2^ = 0.08, whereas all other significant differences in the baseline and experimental tasks remain the same. This demonstrates that the difference between the two groups on their non-verbal abilities did not affect the results of the baseline and experimental tasks.
